# Numerical Analysis of MIM-Based Log-Spiral Rectennas for Efficient Infrared Energy Harvesting

**DOI:** 10.3390/s20247023

**Published:** 2020-12-08

**Authors:** Ali Yahyaoui, Ahmed Elsharabasy, Jawad Yousaf, Hatem Rmili

**Affiliations:** 1 Center of Research Excellence in Renewable Energy and Electrical Power Systems, King Abdulaziz University, P.O. Box 80204, Jeddah 21589, Saudi Arabia; amalyahyaoui@uj.edu.sa; 2 Electrical and Electronic Engineering Department, College of Engineering, University of Jeddah, Jeddah 21589, Saudi Arabia; 3 Electrical and Computer Engineering Department, McMaster University, Hamilton, ON L8S 4K1, Canada; elsharay@mcmaster.ca; 4 Department of Electrical and Computer Engineering, Abu Dhabi University, 1790 Abu Dhabi, UAE; jawad.yousaf@adu.ac.ae

**Keywords:** photoconductive THz antenna, log spiral antenna, MIM rectenna, wideband, high directivity

## Abstract

This work presents the design and analysis of a metal-insulator-metal (MIM)-based optical log spiral rectenna for efficient energy harvesting at 28.3 THz. To maximize the benefits of the enhanced field of the proposed nano-antenna in the rectification process, the proposed design considers the antenna arms (Au) as the electrodes of the rectifying diode and the insulator is placed between the electrode terminals for the compact design of the horizontal MIM rectenna. The rectifier insulator, Al_2_O_3_, was inserted at the hotspot located in the gap between the antennas. A detailed analysis of the effect of different symmetric and asymmetric MIM-configurations (Au-Al_2_O_3_-Ag, Au-Al_2_O_3_-Al, Au-Al_2_O_3_-Cr, Au-Al_2_O_3_-Cu, and Au-Al_2_O_3_-Ti) was conducted. The results of the study suggested that the asymmetric configuration of Au-Al_2_O_3_-Ag provides optimal results. The proposed design benefits from the captured E-field intensity, I-V, resistivity, and responsivity and results in a rectenna that performs efficiently.

## 1. Introduction

Continuing global industrial revolution and increases in the population have led to a rapid increase in the world’s energy requirements [1,2,3,4]. The capacity of conventional energy generation sources is decreasing due to the ongoing decline in fossil fuel reserves [5,6,7]. Renewable energy sources are emerging as a prominent alternative option to produce cheap and clean energy to fulfill our persistent, long-term global energy demands [1,4,8,9,10,11]. 

Conventional solar cell-based photovoltaic (PV) renewable technology is used to harvest the energy from the visible spectrum (400 to 750 nm) of the sun [1,2,5]. However, the major component of solar energy (>50%) lies in the infrared region (IR) (with maximum emissivity at 10.6 µm or 28.3 THz), which is untapped by solar cell-based harvesting sources [2,4,5,9,12,13,14]. Optical nano-rectennas designed for the maximum capture of energy at 10.6 µm have emerged as a good solution for sustainable energy harvesting in the IR region [1,2,4,5,8,12,15,16]. 

**Figure 1:**
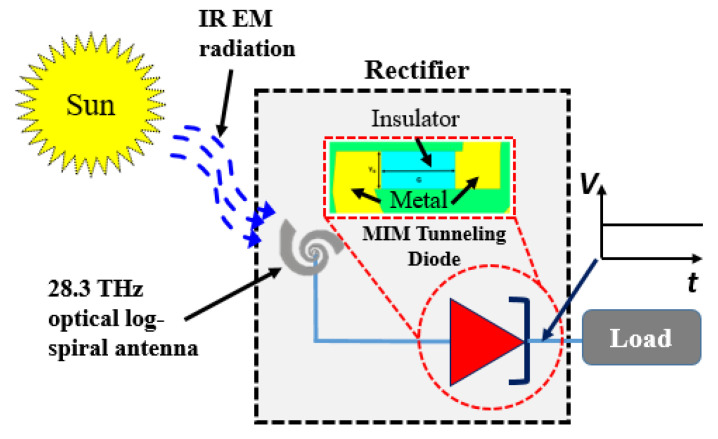
Infrared (IR) rectenna system for energy harvesting.

Figure 1 depicts the general schematic of an infrared rectenna system. The rectenna consists of two main components: an efficient resonant optical antenna at 28.3 THz to capture the maximum IR radiation from the sun, and a tunneling rectifying diode that converts the received IR energy into direct current (DC).

Designing an efficient optimized antenna is vital for the maximum enhancement of the incident solar IR energy [1,3,17,18]. A wide range of nano-antenna structures such as dipole [1,18], bowtie [1,2,4,7,18], slit [6], dimer [19], splitting with two-wire [3], and square patch [17] have been reported for the enhancement of the incident electric field intensity. 

Conventional Schottky diode-based rectifiers are not suitable for rectification at 28.3 THz. The transport time of the electrons across the semiconductor junction is constrained by their drift and diffusion speeds, which limits the working operation of the Schottky diodes up to a few gigahertz. To harvest IR energy at 28.3 THz, special structures based on the tunneling phenomena are used in which electrons can tunnel through a thin layer of oxides packed between two metals [4,11,20,21]. Such structures are referred to as metal-insulator-metal (MIM) diodes [11,14,22,23,24]. The rectification characteristics of such diodes depend on their sensitivity, which is directly proportional to the non-linearity of the diodes I-V properties. For energy harvesting applications, it is crucial that MIM diodes have high sensitivity and low zero-biased resistance without the need for an external bias voltage; also, they must resonate with the nano-antenna to ensure high rectification efficiency [10,11,14,21,23,24]. 

The sensitivity of the MIM diodes depends on the diode structure (symmetric or asymmetric), the metals that are used, the sandwiched oxide insulator(s), the contact area between the antenna’s terminals, oxide thickness, and operating frequency [13,22,25]. Symmetrically structured diodes have the same metals on both sides of the oxide insulator [21,22,24] and vice versa for the asymmetric diode structures [4,8,10,11,13,14,20,23,25,26]. Besides this, as the rectifier has to be integrated with the optical nano-antenna, the value of its zero-bias resistance must be of the same order of magnitude as the designed antenna to ensure the efficient matching and maximum transfer of captured IR energy by the antenna terminal [1,4,6].

A comprehensive overview of nanotechnology and its utilization for IR energy harvesting applications is reported in [27,28,29]. Matsurra et al. [30] proposed an oxygen non-stationary controlled MIIM diode to get high current density and asymmetry. The drawback is the resulting increase in the size of diode. Different kinds of symmetric and asymmetric MIM structures with various coupled antennas (dipole, bowtie, slot, square spiral, Vivaldi, etc.) have been studied in detail in [2,4,10,14,21,22,26,31,32,33,34]. The authors of several studies [30,34,35] have also analyzed the only MIM-based diodes without antenna systems for their potential application in energy harvesting. Recently, the Vivaldi antenna [33]-based MIM rectenna system was proposed for IR energy harvesting. 

**Figure 2:**
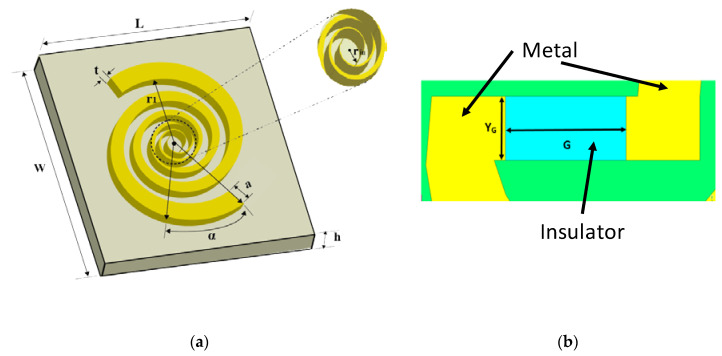
Schematic of the designed rectenna. (**a**) Designed log-spiral nano IR antenna; *r*_1_: inner radius of the spiral, α: increment angle, *a*: growth rate, *t*: thickness of the conductor (gold) material, *L*: length of the substrate, *W*: width of the substrate, and *h*: thickness of the substrate; and (**b**) designed horizontal metal insulator metal (MIM) diode configuration.

This study presents a detailed analysis of the sensitivity of symmetric and asymmetric nano-rectennas. The main goal of our work was to study the effect of different types of metals for the two arms of the proposed horizontal MIM-based rectenna on the rectification and the effect of fixing the insulator Al_2_O_3_ between the feeding point of the antennas (the hotspot region). We studied the E-field, I-V characteristics, resistance, and responsivity for various combinations of rectenna metals. With the results of this study, we can provide guidelines for the configuration that provides the best rectification performance. The first part of the study involved the design of an optimized log-spiral optical antenna (shown in Figure 2) for field enhancement as compared to previously reported antenna structures [1,2,3,4,6,7,17,18,19]. The second part of the study involved the analysis of a wide range of symmetric and asymmetric MM-diodes. The analysis was performed for the following MIM diode configurations: Au-Al_2_O_3_-Ag, Au-Al_2_O_3_-Al, Au-Al_2_O_3_-Cr, Au-Al_2_O_3_-Cu, and Au-Al_2_O_3_-Ti. To maximize the benefits of the enhanced field of the designed nano-antenna in the rectification process, the antenna arms were used as electrodes for the MIM-diode, and the rectifier oxide insulator was inserted at the hotspot location, that is, between the gap of the antenna poles where the maximum electric field is induced (see Figure 2b). This approach helped us to localize the enhanced field immediately over the diodes with the inserted insulator as the main propagation medium, and this increases the coupling efficiency and the rectifier’s output voltage. The study also involved optimizing the rectenna design by finding the ideal materials and oxide thickness, width, and length for obtaining low resistivity, low capacitance, and high non-linear I-V in the realized MIM diode.

The rest of the paper is organized as follows. A comprehensive review of related previous designs is detailed in Section 2. Section 3 describes the proposed log-spiral based rectenna design. The details of the parametric study for the optimized parameters of the realized log-spiral rectenna are given in Section 4. Section 5 presents the analysis of the effects of the change in the MIM diode materials on the performance of an optimized log-spiral rectenna structure. 

## 2. Related Work 

We analyzed the various kinds of MIM-structures that have been used for the efficient design of nano-rectennas to maximize rectification efficiency. Edge et al. [36] analyzed the optical conversion efficiency of three broadband antennas (log-period, square-spiral, and Archimedean-spiral) coupled with Al/Al_2_O_3_/Pt rectifying diodes in the frequency range of 0 to 100 THz. In [37], the authors investigated the performance of the log-spiral nano-rectenna for harvesting blackbody radiation. 

The studies in [21,22] analyzed the performance of a dipole-based rectenna with a symmetric configuration of coupling Ni-NiO-Ni diodes in terms of responsivity and zero-bias resistance. Esfandiari et al. [10] studied the DC characteristics of different symmetric and asymmetric combinations of ultra-small dipole antenna-coupled metal-oxide-metal (MOM)-based structures for the detection of 10.6 µm infrared radiation. Krishnan et al. [20] characterized the performance of Ni-NiO-Cr/Au MIM structures at 2.5 GHz using a microstrip slot antenna. The authors of [14] and [25] reported that the utilization of polysilicon as the metallic material in planar type tunneling diodes enhanced their responsivity. Bean et al. [13] coupled the Al-AlO_x_-Pt diode with a dipole antenna for IR rectification. Zhang [23] studied the operation of a Ni-NiO-Cu diode by varying the thickness of the oxide layer. Zhu et al. [24] investigated the performance of a bow-tie-based rectenna by integrating a graphene-based MIM diode to achieve higher zero-bias resistance at 28 THz. The geometric diode structure presented in [24] provides advantages over parallel plate diodes in terms of RC constrains. The current distribution and input resistance of wire nano-rectennas using integral equations modeling are presented in [31]. 

Recent studies [2,4,32] have characterized the performance of asymmetric MIM rectennas in terms of the enhancement in the captured E-field and DC analysis by placing the MIM diode at the antenna feeding points. This work characterizes the E-field enhancement and DC performance of various ultra-small symmetric and asymmetric horizontal MIM-rectennas using a different log-spiral antenna for IR energy harvesting. Table 1 summarizes a comparison of the proposed work with previous designs.

## 3. Rectenna Design

In this work, we used a wideband log spiral nano-antenna to capture the incoming infrared RF energy from the sun. Figure 2a shows the design of nano-antenna for the proposed energy harvesting system. The wideband frequency and circularly polarized operation of such antennas are obtained by the gradual opening of the arms and slots of the antenna and the feeding of the antenna source [38,39,40]. The fundamental equation to describe the relationship between the *nth*- turn of the spiral antenna and other design parameters is shown in Equation (1).

(1) $$r_{n}=r_{o}e^{\left(a\left(\alpha +\varphi_{n}\right)\right)}$$

The maximum angle of the nth radius is defined by $\varphi_{n}$ while the α represents the initial angle at the origin of the spiral turn. The parameter *a* determines the growth rate of spiral turns [40,41]. The high frequency operation of the antenna is limited by the inner radius (*r*_1_). 

An extensive parametric study of all of the parameters of the antenna shown in Figure 2 was conducted to find the optimized dimensions for the maximum capture of the incident electric field in the frequency range of 28 THz to 29 THz. The details of the parametric study can be found in [42]. Table 2 shows the optimized design parameters of the nano-antenna. The substrate material of the designed antenna is quartz (${}_{r}$ = 3.78, tan δ = 0.0001) while gold (Au) was used as the printed conductor material on the substrate. We used the quartz because of its low-loss characteristics and because it is better for capturing of the incident electric field, which is necessary for operation in the high frequency range [43]. Gold was chosen as the conductor material because of the refractive index of the material. A gold thickness of 62 nm was obtained after the structure was optimized to achieve the required behavior and also by taking the fabrication constraints into consideration. All simulations are performed in CST (Computer Simulation Technology)-Microwave Studio software. The designed antenna shows resonance at 28.3 GHz, which is the key for the field enhancement of incident solar radiation at 10.6 µm (the peak of the earth’s emissivity). 

After the design of the antenna terminal was completed, a horizontal MIM-based diode was designed and integrated at the antenna terminals as shown in Figure 2b. The design variables of the MIM structures are its contact area (the width and length of the insulator), insulator thickness, and the metals use on both sides of the insulator [29]. In Figure 2b, G defines the length of the gap while Y_G_ represents the width of the antenna terminals in the corresponding gap. The thickness of the insulator between the two arms is the same as the thickness of the metal, i.e., 62 nm. Matching the rectifier antenna and diode requires low input impedance, which requires a large contact area [13,20,21]. This reduces the cutoff frequency due to the increased capacitance and also enhances the response time of the diode. Similarly, the increase in the oxide thickness enhances the sensitivity of the diode due to the increase in the non-linear characteristics of the diode [4,8,10,23]. However, careful analysis of this increase in the oxide thickness is required as the slide increase in the thickness of the used oxide layer could drastically increase the diode’s resistance, and thus impact the antenna-diode matching [4,8,13,22,25].

For the maximum coupling efficiency, instead of integrating a separate diode with the receiving antenna, we used the antenna arms as rectifying diode metals and inserted the thin insulator (oxide) layer in-between the gap of the antenna port. The utilization of this strategy of inserting the insulator in the hotspot region (the feeding point of the antenna), as illustrated in Figure 2b, forms a horizontal MIM configuration that helps the antenna to maximize the benefit of the enhanced field and minimize the transfer loss. This approach maximizes propagation through the insulation junction, and thus enhances the levels of the rectified output. 

The chosen metal also has a crucial role in the MIM design as it impacts the diode resistance, and thus, its coupling efficiency [11,20,21,23,24]. The use of the metals with different work functions (asymmetric structure) as the antenna arms could be used for higher sensitivity and zero biased rectification. However, the asymmetric design decreases the coupling efficiency of the rectenna due to increasing diode resistance [8,13,14,25]. On the contrary, the symmetric design (with the same metal arms) minimizes the diode resistance but decreases the ability of the diode to work at zero bias [22,24]. 

To meet the aforementioned criterion and trade-offs between the diode contact area and thickness of the diode, different materials with different work functions were used to construct the MIM rectifier. Firstly, a detailed parametric study of the length and width of the insulator material was carried out with a symmetric MIM design. For this purpose, the chosen insulator was Al_2_O_3_ and gold was used as the metal arms of the antenna. We chose Al_2_O_3_ as the insulator because it has a low dielectric constant at THz frequencies, which allows us to match the operational cutoff frequency of 28.3 THz for the maximum capture of the incident IR radiation. After that, a detailed comparative study of various high-doped semiconductor materials was carried out to find the optimal design choices to satisfy the requirements of the diode at zero bias for low resistance, low capacitance, and high-non-linearity.

## 4. Parametric Study

In this work, we used the CST (Computer Simulation Technology)-Microwave Studio software package, which is based on the three-dimensional full-wave electromagnetic field finite integral technique (FIT). The FIT is a consistent discretization scheme for Maxwell’s equations and it provides a spatial discretization scheme that is applicable to several electromagnetic problems. We designed a rectenna for operation at 28.3 THz frequency and by assuming an open space in the x-, y- and z- directions as the boundary conditions. The structure (Figure 2) is composed of a spiral gold shape whereas the substrate is made of quartz with permittivity equal to 3.78. To excite the proposed structure, we used a plan wave excitation to excite the gap (*G*), i.e., between the two arms of the spiral antenna. A parametric analysis was performed to obtain the optimized design parameters (length of the insulator (*G*) and gap of the insulator (Y_G_)) to collect the maximum E-field. In addition, we used the Drude model to describe the transport properties of electrons in the conductor at this range of frequencies by respecting the realizable dimensions and by taking into consideration the possibility of fabrication in the future. After optimizing the different parameters of the rectenna to obtain the maximum electric field, we added the insulator (Au-Al_2_O_3_-Au.) to increase the output voltage of the rectifier, that is, to increase the efficiency. The performed simulation was done using a professional workstation with high performance (16 CPU @ 2.6 GHz with 64 GB of RAM), and the simulation of each iteration took at least 20 minutes.

### 4.1. Variation in the Length of the Gap (G)

**Figure 3:**
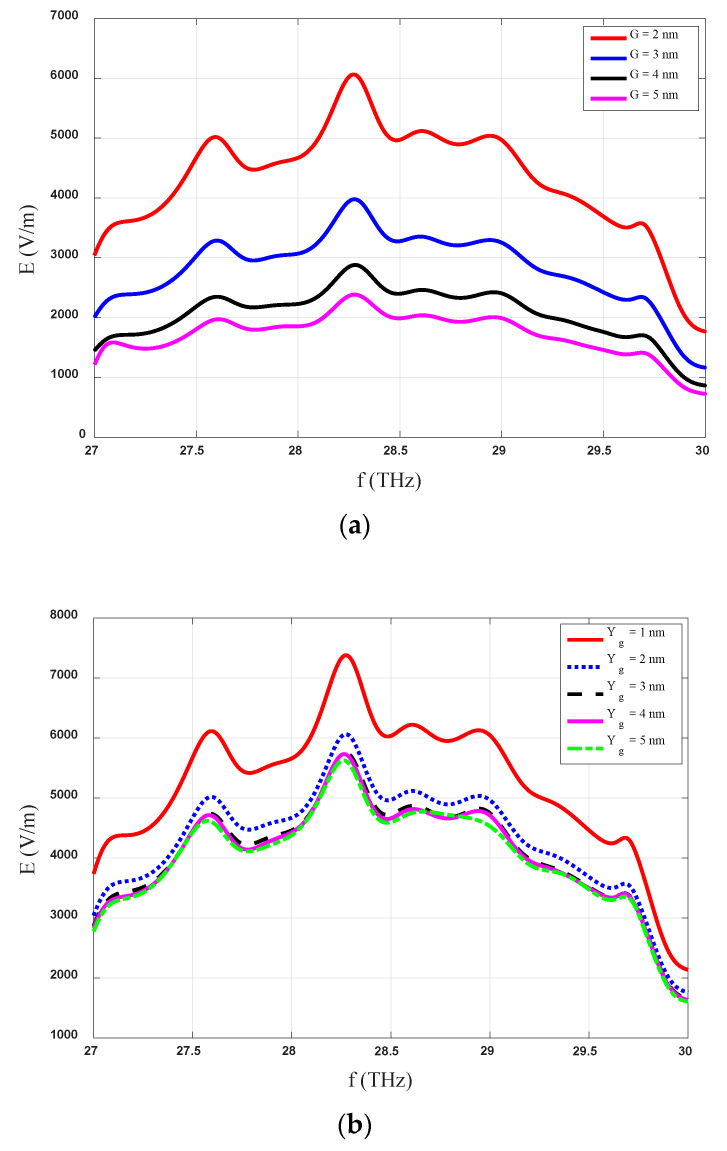
Effect of MIM dimensions (**a**) gap length (G) and (**b**) width (Yg) of insulator on the induced electric field.

Figure 3a shows the variations in the level of the induced electric field across the gap region with the change in the gap length values. The analysis was performed for variations in the gap ranging from 2 nm to 5 nm with a step size of 1 nm. The maximum recorded E-field level with a gap length of 2 nm, 3 nm, 4 nm, and 5 nm was around 6000 V/m, 400 V/m, 2900 V/m, and 2250 V/m, respectively. Notably, the maximum levels of the induced field are observed with a minimum gap length of 2 nm at the resonance frequency of the designed MIM, i.e., 28.3 THz. The inductance of the diode decreases with the decrease in the gap width, which results in the enhancement of the field levels as shown in Figure 3a. The induced E-field levels decrease with the increase in the gap length due to the enhancement of the inductance of the gap region.

### 4.2. Varying the Gap Width ($Y_{G}$)

The role of the gap width between the electrodes (the width of the insulator in our case) of the rectenna is vital for the absorption of maximum incident solar radiation. The effect of changing the width of the insulator on the induced E-field between the gap of the antenna arms (electrodes) is depicted in Figure 3b. The maximum induced levels are observed with the smallest value of the *Y_G,_* i.e., 1 nm while the field levels decrease with the increase in the length of the insulator material. 

The thicknesses of the insulating layers affect the diode capacitance, which determines its cutoff frequency. These trade-offs affect the performance of the rectenna (degradation), therefore we should optimize all the geometrical parameters including the width of the insulator. The increase in the width of the insulator decreases the capacitance of the structure, and thus results in the lower values of the induced electric field. The captured E-field levels have significant variations with a higher induced E-field when the insulator width is reduced to lower levels. Although the insulator gap of 1 nm produced the best results, the manufacturing of such a structure could be difficult due to limitations in fabrication facilities. For this reason, larger insulator gaps (4 nm or 5 nm) could be chosen as the optimal value while keeping in mind the fabrication limitations because variations in the gap width from 2 nm to 5 nm did not cause any significant change in the captured levels of incident IR radiation. 

### 4.3. Analysis of the Optimized Structure

**Figure 4:**
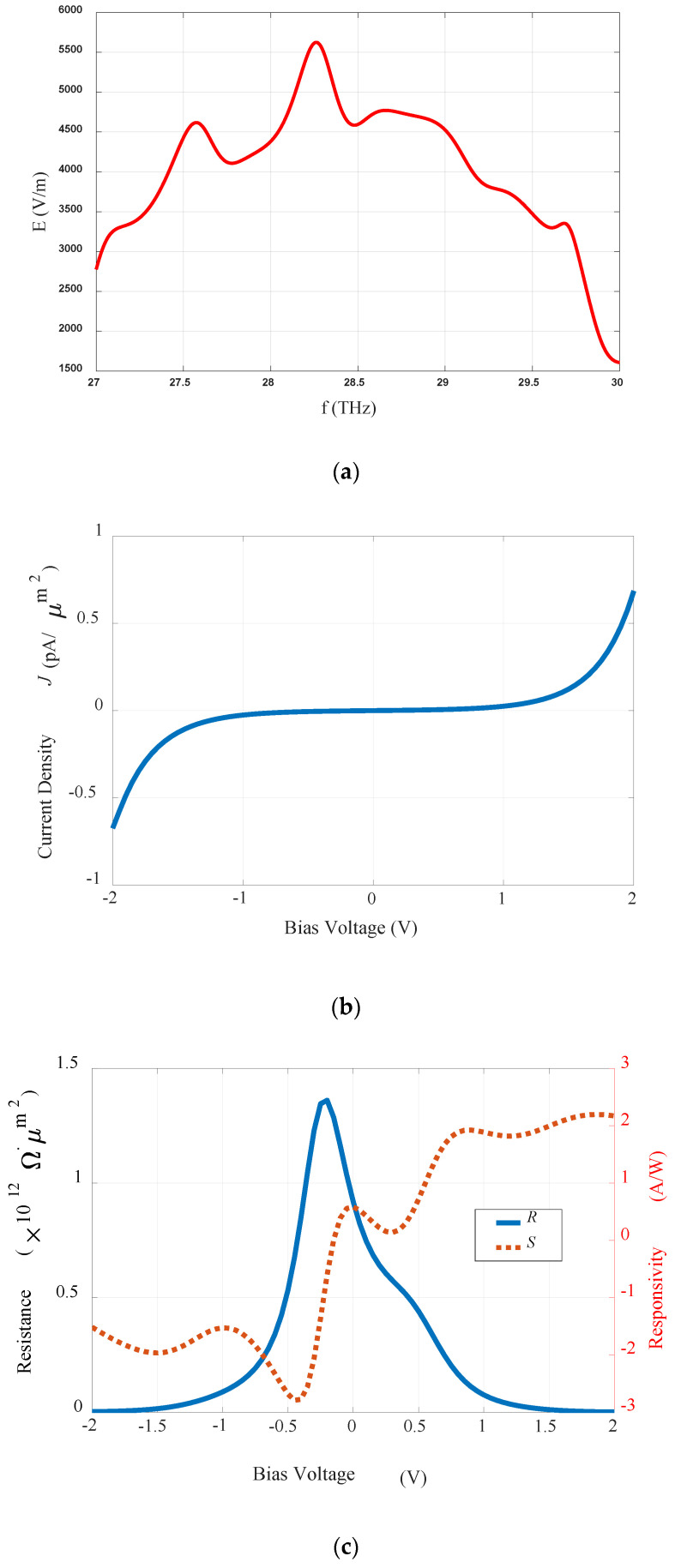
Characteristics of the optimized Au-Al_2_O_3_-Au log spiral rectenna: (**a**) induced electric field; (**b**) current-voltage; and (**c**) resistance and responsivity.

The variations in the induced electric field level of the proposed optimized MIM-based log spiral rectenna with frequency are shown in Figure 4a. These results are for the optimized insulator (Al_2_O_3_) with dimensions of *G* = 2 nm and *Y_G_* = 5 nm. The gap size (G) between the two arms of the proposed structure has a major impact on the behavior of the proposed rectifier system in the real environment. The dimensions of the optimized structure were chosen while keeping in mind the constraints of a real-time environment structure and the limitations of actual fabrication. The optimized value of the length of the oxide gap (*G* = 2 nm) was obtained from the parametric analysis shown in Figure 3a, where the maximum level of the induced E-field was found for this insulator gap length. On the contrary, instead of choosing Y_G_ = 1 nm as the optimal value of the width of the oxide gap from Figure 3b, the value of 5 nm was selected as this value is most suitable for the practical realization of the proposed rectenna, keeping in mind the limitations of the fabrications process. Along with the parametric analysis, in this work we finalized the G and Yg values based on the experimental difficulties noted in previous research [8,35].

It can also be seen in Figure 3b that the change in the width of the gap from 1 nm to higher values (2–5 nm) reduces the value of the induced E-field, however, the captured E-field level is in the range of 6000 V/m, which is a good for capturing IR radiation. Also, the change in the Yg from 2 nm to 5 nm had an almost negligible impact on the captured E-field levels. Therefore, a 5 nm gap width was chosen for the insulator as the optimized value of the proposed rectenna because it meets the fabrication constraints. 

The results of the current-voltage characterization of the optimized Au-Al_2_O_3_-Au log spiral rectenna are depicted in Figure 4b. The DC characterization of the developed rectenna was done by computing the resistance and responsivity of the diode. The definition of the diode’s responsivity and resistance (S and R) can be described at zero-bias by the following equations [8].

(2) $$R=\frac{1}{I^{\prime }}$$

(3) $$S=\frac{I^{\prime \prime }}{2I^{\prime }}=\frac{R.I^{\prime \prime }}{2}$$

In Equations (2) and (3), I′ is the first derivative of the current with respect to the voltage while I″ represents the second derivative of the current with respect to the voltage. 

The noticeably symmetric behavior between the current-voltage characteristics of the proposed rectenna can be observed in Figure 4b when the DC bias voltage varies in the range of −2 V to +2 V. The resistance and responsivity of the proposed rectenna are shown in Figure 4c. In Figure 4c it is notable that the Au-Al_2_O_3_-Au tunneling diode has a zero-biased resistance and responsivity of 0.9 × 1012 Ω.µm2 and 0.5 A/W, respectively. The maximum obtained values for the resistance and responsivity at the resonance frequency of 28.3 THz are 1.47 × 1012 Ω.µm2 @ −0.1 V and 1.85 @ 2 V, respectively, as shown in Figure 4c.

## 5. Study of the Material Effects on the Optimized Log-Spiral Rectenna Structure

This section presents a detailed study of the effect of the different materials of the proposed log-spiral rectenna on the induced E-field levels, current-voltage, resistance and responsivity. All of these characteristics were analyzed for the five different cases of the MIM-diode metallic materials, that is, Au-Al_2_O_3_-Ag, Au-Al_2_O_3_-Al, Au-Al_2_O_3_-Cr, Au-Al_2_O_3_-Cu, and Au-Al_2_O_3_-Ti. Using the same insulator, i.e., Al_2_O_3_, the material on the one side of the log-spiral antenna (Figure 2) was fixed as gold (Au) while the other side was changed to silver (Ag), aluminum (Al), chromium (Cr), copper (Cu) and titanium (Ti) for the case study analysis. The results for each case were compared with the base case of Au-Al_2_O_3_-Au. The work function of all the analyzed metals are shown in Table 3 [12].

### 5.1. E-Field 

**Figure 5:**
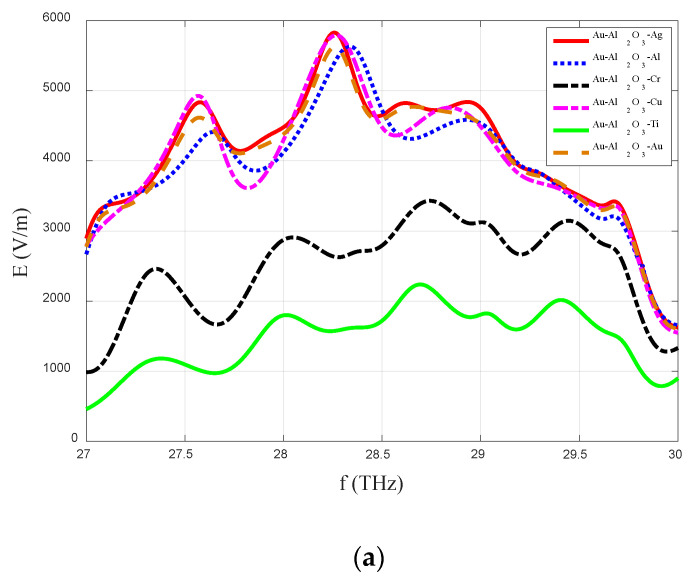

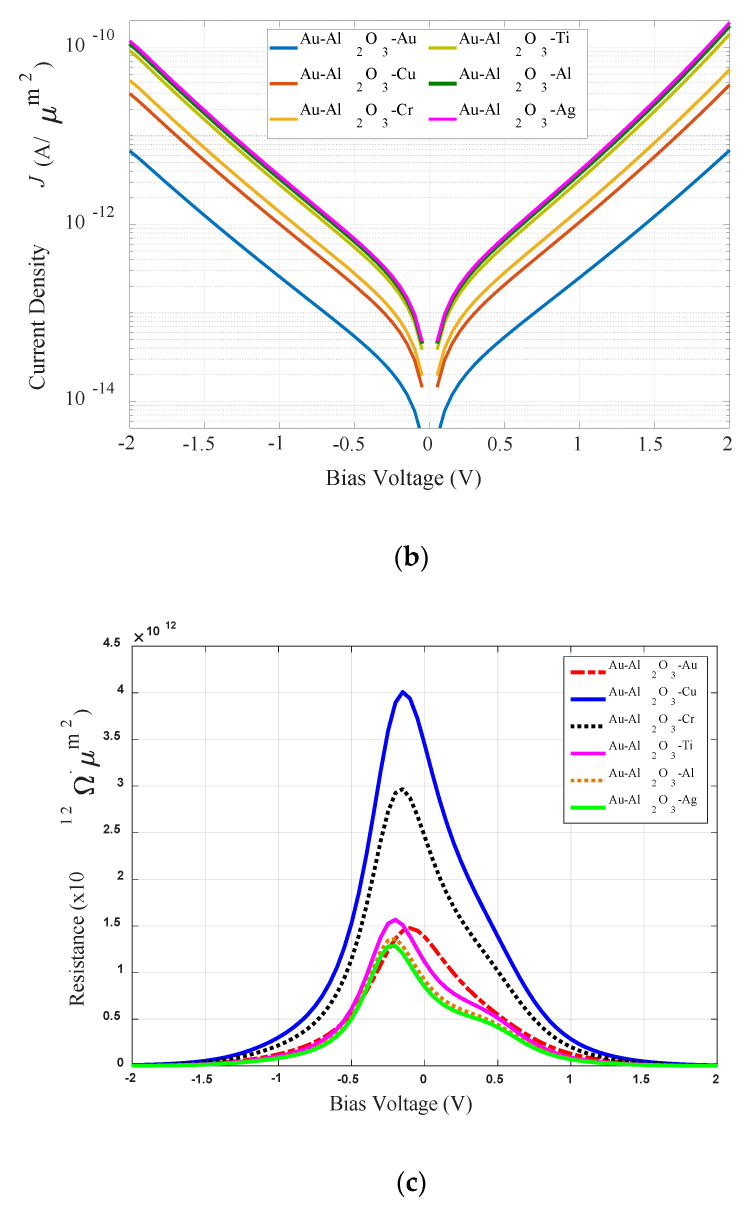
Variations in (**a**) the induced electric field, (**b**) the current-voltage characteristics, and (**c**) the resistance characteristics with the different material configurations of the MIM-diode.

The impact of the MIM-diode metallic materials on the field enhancement of the proposed log-spiral rectenna was analyzed for the six different aforementioned cases. The induced E-field levels for the MIM-diode configurations of Au-Al_2_O_3_-Ag, Au-Al_2_O_3_-Al, Au-Al_2_O_3_-Cr, Au-Al_2_O_3_-Cu, Au-Al_2_O_3_-Ti, and Au-Al_2_O_3_-Au are shown in Figure 5a. In Figure 5a, the maximum levels for the field enhancement can be observed for the MIM-diode configurations of Au-Al_2_O_3_-Ag, Au-Al_2_O_3_-Al, Au-Al_2_O_3_-Cu, and Au-Al_2_O_3_-Au_._

The lower values for the induced E-field in the cases of the titanium (Ti) and chromium (Cr) could be due to the smaller difference in the work function values of these metals with the fixed metal of Au, which impacts the electron tunneling, and hence the field enhancement levels. For the remaining cases of Ag, Al, Cu, and Au, the field enhancement levels are almost invariant with the change in metal. Also, it can be observed in Figure 5a that the maximum level of field enhancement is at the resonance frequency of 28.3 THz.

### 5.2. Current-Voltage Characteristics

The variations in the current-voltage characteristics of the different configurations of the MIM-diode are depicted in Figure 5b. As expected, the best results were obtained for the Au-Al_2_O_3_-Ag case as the electron tunneling is highest for this case because of the maximum difference in the work function of these metals (see Table 2). The lowest level was observed for the Au-Al_2_O_3_-Au case because there is zero difference in the work function of the metals on both sides of the log-spiral antenna arms. Higher current-voltage levels are obtained when the difference between the work functions of the used metals is increased, which has a direct impact on the electron tunneling of the MIM diode configuration.

### 5.3. Resistance Characteristics

Figure 5c presents the variation in the resistivity of the MIM-diodes for the analyzed metals. The results show that the maximum and minimum values of resistance are obtained for the Au-Al_2_O_3_-Cu and Au-Al_2_O_3_-Ag configurations, respectively. Table 3 summarizes the maximum resistivity values of the Figure 5c curves.

### 5.4. Responsivity Characteristics

**Figure 6:**
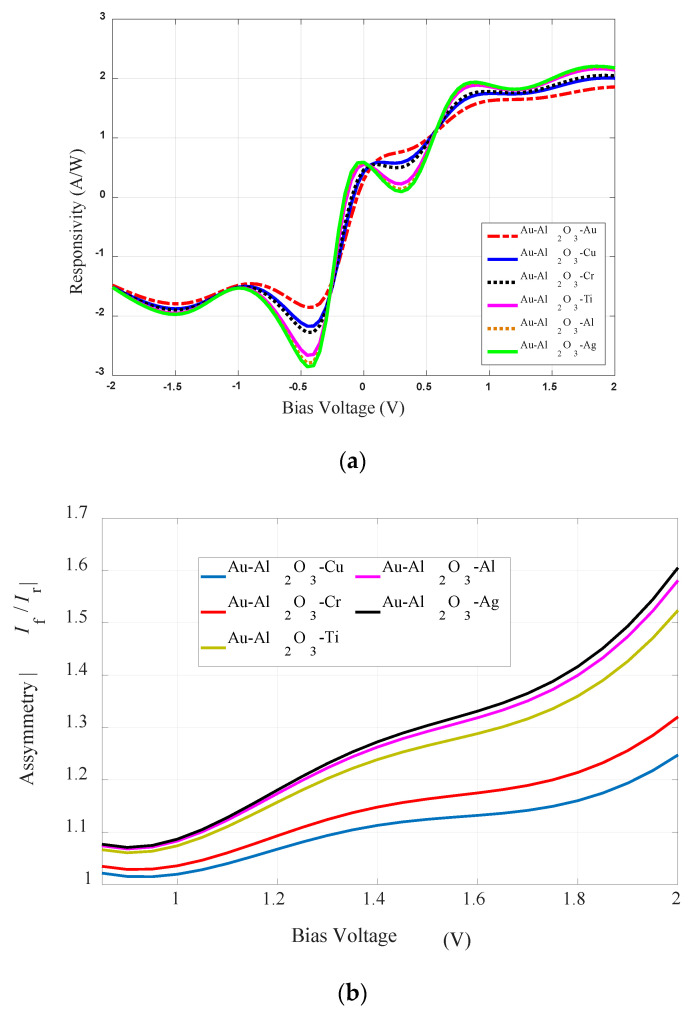
Variations in (**a**) the responsivity and (**b**) the symmetry characteristics with the different material configurations of the MIM-diode.

The change in the responsivity properties of the analyzed diodes is depicted in Figure 6a while Table 3 presents the maximum values of the responsivity of each case shown in Figure 6a. Figure 6a and Table 4 show that the Au-Al_2_O_3_-Ag diode has the maximum value (2.2 A/W @ 2 V) of *S*. The Au-Al_2_O_3_-Au diode has the minimum value of responsivity of 1.85 A/W @ 2 V. However, as can be noted from Figure 6a, the responsivity characteristics of the diodes with different metals are relatively invariant as compared to the resistivity properties (Figure 5c) of the diodes. 

### 5.5. MIM-Diodes’ Asymmetry

Figure 6b shows the asymmetry characteristics of the different analyzed diodes. Although the trend in asymmetry was similar for all diodes, i.e., it increased with the increase in the bias voltage level from 0 V to 2 V, the maximum value of asymmetry was observed for the Au-Al_2_O_3_-Ag diode. The resistivity and responsivity obtained for the analyzed symmetric and asymmetric rectenna configurations make it a good choice for a rectenna for IR energy harvesting at 28.3 THz. 

The main disadvantage or limitation of IR rectenna systems for energy harvesting is related to the fabrication of rectifiers, which depends on the limits of nanotechnology. The current nanofabrication technology is still not mature enough to fabricate high performance rectifiers, especially in regard to efficiency and thickness. Even though the fabrication of the proposed structure is possible in a laboratory, its mass fabrication and integration into a large array configuration are challenges for the actual implementation of IR rectenna systems.

## 6. Conclusions

This study has discussed the design and analysis of a horizontal metal-insulator-metal (MIM)-based optical log spiral rectenna for efficient energy harvesting at 10.6 µm. We designed an efficient rectenna, which constitutes an optimized log-spiral nano-antenna, to ensure maximum coupling efficiency and instead of integrating a separate diode with the receiving antenna, we used the antenna arms as rectifying diode electrodes and inserted the thin insulator (oxide) layer in-between the gap of the antenna port. A comprehensive analysis of the effect of the various symmetric and asymmetric MIM-configurations suggested that the use of the asymmetric MIM of Au-Al_2_O_3_-Ag with a log-spiral antenna provides the best results for enhancing incident IR solar energy intensity and the higher conversion efficiency of the realized rectenna. 

The design strategies that were adopted ensure that the proposed solution is suitable for the future fabrication of a real system and the measurable benefits of the proposed design strategy can be obtained in various applications. The determination of the various rectenna design parameters such as the oxide thickness, metal thickness per production constraints, and the minimum linear dimension that is compatible with optical and/or e-beam lithography, etc., makes the presented approach useful from a manufacturing point of view. The presented design could be fabricated for potential application in energy harvesting.

## Figures and Tables

**Table 1:** Comparison of the proposed design with previous designs.

Ref.	Diode Configuration	Nano-Antenna	Operating Frequency (THz)	Maximum Responsivity (V-1)	Zero-Bias Responsivity (V-1)	Zero-Bias Resistance (ohm)
[21]	Ni-NiO-Ni	Dipole	30	1.6	--	100
[22]	Ni-NiO-Ni	Dipole	0.09/28	2.75/1.65	0	180
[10]	Al- Al_2_O_3_-Al/ Al- Al_2_O_3_-Ti/ Al- Al_2_O_3_-Pt/ Al- Al_2_O_3_-Ni/Ni-NiO-Pt	Dipole	28.3	−1.4 /2/1.3/1/−13	0.1/0.6/2/0.5/−3	10 M/-/-/-/10 M
[20]	Ni-NiO-cr/Au	Microstrip slot	0.0025	5	1	500 K
[25]	polysilicon-SiO2-polysilicon	Bow-tie	-	−31	12	--
[14]	polysilicon-SiO2-Au	Bow-tie	0.005	−14.5	2.5	120 M
[13]	Al-AlOx-Pt	Dipole	28.3	−2.3	0.5	220 K
[23]	Ni-NiO-Cu	-	-	7.3	7	1.2 M
[11]	Al- Al_2_O_3_-Pt	Slot antenna based FSS	28.3	0.03	1.24 × 10^−3^	124.6
[24]	Graphene-Air-Graphene	Bow-tie	28	0.24	0.12	1 K
[31]	Au-*-Au	dipole	10–90	-	-	-
[2]	Cu-CuO-Au	Bowtie	28.3	6	-	505
[26]	Au-TiOx-Ti	Dipole	30	1 × 10^−4^	-	440 M
[32]	Au-TiOx-Ti	Square spiral	28.3	0.3	−0.44	--
[4]	Au- Al_2_O_3_-Ag	Bowtie	28.3	1.25	0.44	98 K
[30]	Pt-TiO_2_--TiO_1,4-_Ti	-	-	4.6 × 10^6^	4 × 10^3^	-
[34]	Al-AlOx-Gr	-	-	2.5 × 10^3^	-	600
[35]	Ti-TiO2-Al	-	-	3.1	0.15	275
[33]	Au- Al_2_O_3_-Ti	Vivaldi	28.3	2.5	1	4 × 10^11^
This work	Au- Al_2_O_3_-Au/ Au- Al_2_O_3_-Cu/ Au- Al_2_O_3_-Cr/ Au- Al_2_O_3_-Ti/ Au- Al_2_O_3_-Al/ Au- Al_2_O_3_-Ag	Log-spiral	28.3	1.85/2.01/2.05/2.16/2.19/2.2	0.5/0.5/05/0.5/0.5/0.5	1.4/3.52.5/1.2/0.9/0.85 E12

**Table 2:** Designed nano-IR antenna parameters.

Parameter	Values(µm)
*L* (substrate length)	7.8
*W* (substrate width)	7.8
*h* (substrate thickness)	99.1
*r*_1_ (inner radius of the spiral)	0.02
*N* (number of turns)	2.1
*δ* (spiral-patch width)	115
*α* (increment angle)	1.8°
*a* (growth rate)	0.31
*t* (spiral-patch thickness)	0.062

**Table 3:** The work function of the analyzed metals.

Metal	Work Function (eV)	Work Function Difference (eV) with Respect to Au
Gold (Au)	5.1	0
Silver (Ag)	4.26	0.84
Aluminum (Al)	4.28	0.82
Chromium (Cr)	4.5	0.60
Copper (Cu)	4.53/5.1	0.57/0
Titanium (Ti)	4.33	0.77

**Table 4:** Maximum resistance (R) and responsivity (S) for the different metals of the MIM-diode at 28.3 THz.

Configuration	Max R (×10^12^ Ω.µm^2^)	Max S (A/W)
Au- Al_2_O_3_-Au	1.47 @ −0.1 V	1.85 @ 2 V
Au- Al_2_O_3_-Cu	4 @ −0.15 V	2.01 @ 2 V
Au- Al_2_O_3_-Cr	2.96 @ −0.15 V	2.05 @ 2 V
Au- Al_2_O_3_-Ti	1.56 @ −0.2 V	2.16 @ 2 V
Au- Al_2_O_3_-Al	1.36 @ −0.2 V	2.19 @ 2 V
Au-Al_2_O_3_-Ag	1.28 @ −0.25 V	2.2 @ 2 V
